# MyLungHealth, a Patient-Facing Education Tool for Lung Cancer Screening: Qualitative User-Centered Design Study

**DOI:** 10.2196/83033

**Published:** 2026-05-21

**Authors:** Christian Andrew Balbin, Leticia Stevens, Rachel Dalrymple, Victoria L Tiase, Kimberly A Kaphingst, Elizabeth R Stevens, Polina V Kukhareva, Tanner J Caverly, Guilherme Del Fiol, Devin Mann, JaeEun Kwon, Angela Fagerlin, Jorie M Butler, Kensaku Kawamoto

**Affiliations:** 1 Department of Biomedical Informatics, University of Utah, 421 Wakara Way #140, Salt Lake City, UT, 84108, United States, 1 (801) 581-4080; 2 Department of Communication, University of Utah, Salt Lake City, UT, United States; 3 Huntsman Cancer Institute, University of Utah, Salt Lake City, UT, United States; 4 Department of Population Health, NYU Grossman School of Medicine, New York City, NY, United States; 5 Department of Health Informatics, NYU Langone Health, New York City, NY, United States; 6 Department of Learning Health Sciences, University of Michigan, Ann Arbor, MI, United States; 7 Center for Clinical Management Research, Department of Veterans Affairs, Ann Arbor, MI, United States; 8 Department of Population Health Sciences, University of Utah, Salt Lake City, UT, United States; 9 Informatics, Decision-Enhancement, and Analytic Sciences (IDEAS) Center of Innovation, VA Salt Lake City Health Care System, Salt Lake City, UT, United States; 10 Department of Internal Medicine, Division of Geriatrics, University of Utah School of Medicine, Salt Lake City, UT, United States; 11 VA Salt Lake City Geriatric Research, Education, and Clinical Center (GRECC), Salt Lake City, UT, United States

**Keywords:** lung cancer screening, low-dose computed tomography, shared decision-making, patient decision aid, user-centered design, electronic health record integration, patient portal, personal health record, personalized risk communication

## Abstract

**Background:**

Lung cancer remains the leading cause of cancer-related mortality worldwide, with low-dose computed tomography screening demonstrating an approximately 20% reduction in mortality among high-risk individuals. Despite this benefit, screening prevalence remains suboptimal, with often less than 20% of eligible individuals reported to be up to date on screening. Shared decision-making is essential for effective lung cancer screening (LCS) implementation, with decision aids shown to enhance patient knowledge and engagement.

**Objective:**

The aim of this study is to identify patient preferences, concerns, and design considerations through qualitative evaluation of MyLungHealth, a personalized patient-facing educational tool for LCS integrated with electronic health records, and to describe how these findings informed iterative design modifications.

**Methods:**

We employed qualitative research methods through focus groups (n=34) and individual interviews (n=18) with individuals who met screening eligibility criteria. Participants were recruited from the University of Utah Health and New York University Langone Health between May and December 2023. Feedback was analyzed using Braun and Clarke’s thematic analysis principles.

**Results:**

Six themes were organized into three overarching domains. Domain A included interpretation and impact of personalized risk information: theme 1, difficulties interpreting risk information, and theme 2, varied impacts of risk information on motivation. Domain B included autonomy, privacy, and user interface preferences: theme 3, desire for autonomy and control over personal health data, and theme 4, preference for straightforward language and multiple information formats. Domain C included integration with clinical workflows and patient portal systems: theme 5, expectations for integration with health care provider workflows, and theme 6, mixed experiences with personal health record systems. These insights led to key design modifications, including simplified risk presentation, multimodal content delivery options (video and text), and implementation of electronic health record alerts for clinicians.

**Conclusions:**

The user-centered design process for MyLungHealth revealed important considerations for developing effective patient education tools for LCS. The findings highlighted the need for simplified risk presentation, personalized information delivery, and integration with clinical workflows. These findings underscore the importance of balancing comprehensive risk communication with user accessibility.

## Introduction

Lung cancer remains the leading cause of cancer-related mortality in the United States and worldwide [1,2]. Low-dose computed tomography (LDCT) screening has demonstrated an approximately 20% reduction in mortality among current and former smokers [3,4]. Despite this benefit, lung cancer screening (LCS) uptake remains suboptimal, with often less than 20% of eligible individuals up-to-date on screening [5]. Shared decision-making (SDM), a collaborative process wherein health care providers and patients make informed decisions aligned with patients’ values and preferences [6], is widely considered a critical component of LCS implementation. In the United States, the Centers for Medicare & Medicaid Services requires a documented SDM visit using a decision aid before reimbursement for LDCT screening [7]. SDM is particularly relevant in LCS due to the considerable variability in risk-benefit profiles among eligible patients and the need to balance potential benefits against risks, such as false positives that may lead to unnecessary invasive procedures [4].

A Cochrane systematic review of over 200 studies found that decision aids used for people facing health treatment or choosing whether or not to be screened for various diseases significantly enhance patients’ knowledge of available options, improve risk comprehension, strengthen alignment between values and choices, and reduce decisional conflicts [8]. Patients using decision aids report feeling more informed and demonstrate increased engagement in the decision-making process [8]. Specifically for LCS, a systematic review published in *Chest* confirmed that decision aids improved patient knowledge and reduced decisional conflict, while receiving high acceptability ratings from both patients and providers [9]. A focus group study published in the *Annals of the American Thoracic Society* revealed that the patients generally desired more comprehensive information regarding the benefits and harms of LCS, found decision aids valuable for making more informed decisions, and preferred that this information be personalized to their specific circumstances [10]. Separately, previous research conducted by our group demonstrated that patients generally seek additional information about LCS prior to clinical visits and support the development of personalized decision aids integrated into the patient portal [11].

Various LCS decision aids exist in multiple formats (print [10,12-16], video [15,17-20], and web [13,21-25]) for use either during clinical encounters or as preparatory tools before visits [9]. However, there remains a gap in leveraging recent advances in federal health data interoperability standards to deliver highly personalized risk assessments based on relevant factors, such as smoking history and comorbidity data from electronic health records (EHRs).

Our research team previously developed Decision Precision+, an EHR-integrated version of the web-based, provider-facing decision aid known as Decision Precision or ScreenLC [26,27]. Decision Precision+ can be accessed within EHRs and automatically retrieves patient data to reduce manual data entry and support clinicians in SDM discussions about LCS with their patients [26,27]. The implementation of the Decision Precision+ tool along with provider-facing prompts to consider LCS was associated with a significant increase in screening rates from 6.8% to 22.8% (adjusted odds ratio 3.6, *P*<.001) among University of Utah Health (UHealth) primary care patients [28]. The subsequent addition of simple patient-facing prompts in the personal health record (PHR) to consider LCS modestly increased screening rates [28]. However, even with these various interventions, the majority of eligible patients did not receive LCS, indicating a need for additional interventions, such as more intensive patient-facing interventions focused on education and empowerment [28].

To address this need, we developed MyLungHealth, a patient-facing educational tool integrated into the PHR, designed to provide personalized LCS information outside of clinical settings. This study employed qualitative research methods to guide the user-centered design (UCD) of MyLungHealth.

This study reports on (1) patient preferences, concerns, and design considerations that emerged during the UCD of MyLungHealth and (2) the resulting design modifications made to optimize the tool for patient engagement in LCS decision-making. The findings provide valuable insights for optimizing patient-facing decision aids for LCS and potentially other preventive health interventions.

## Methods

### Overview

Following the International Patient Decision Aid Standards recommendations [29] for systematic user-centered development, we used UCD methodologies throughout the development process. This approach aligns with current best practices; a recent systematic review of 607 development studies found a widespread adoption of UCD principles, typically involving multiple rounds of patient feedback and prototyping [30]. UCD maximizes intervention usability for the target population by ensuring alignment with users’ needs, values, and preferred contexts [31,32], while improving satisfaction, engagement, and adoption rates [33].

MyLungHealth is a web application distributed through previsit questionnaires in the patient portal in preparation for SDM in upcoming primary care appointments. It provides educational content on LDCT, personalized risk assessments, and comprehensive information on screening benefits and risks in both text and video formats. The initial design was informed by previously identified information needs [11], with subsequent iterative refinement based on feedback from focus groups and interviews. The clinical impact of the resulting intervention was evaluated through a multisite randomized controlled trial at UHealth and New York University Langone Health (NYULH) [34].

We conducted focus groups and individual interviews to gather comprehensive user feedback through an iterative development process. Focus groups and interviews followed a semistructured format, meaning sessions used a standardized discussion guide with prepared questions while allowing flexibility for participants to raise additional topics and for the moderator to probe emerging issues.

The collected data were analyzed using Braun and Clarke’s principles for thematic analysis [35,36]. A multidisciplinary team of 4 researchers experienced in qualitative analyses engaged iteratively with the data to generate codes and develop themes through interpretive discussion and analytic reflection. Consistent with the reflexive thematic analysis, themes were conceptualized as analytic interpretations developed through researcher engagement with participant accounts rather than as preexisting categories awaiting discovery.

To enhance analytic rigor and transparency, we attended to multiple dimensions of trustworthiness throughout the analysis. Credibility was supported through prolonged engagement with the data, regular analytic debriefings, and iterative prototype revisions that functioned as a pragmatic form of member engagement, whereby updated prototypes were presented to subsequent participant groups. Dependability was established through the systematic documentation of coding decisions and the evolution of the analytic framework. Confirmability was supported by a hybrid deductive-inductive approach that anchored analysis in an existing conceptual framework while remaining open to emergent insights, alongside team-based analytic discussions that refined interpretations and thematic structures. Transferability was facilitated through a detailed description of the study context, participant characteristics, and methodological procedures across 2 geographically and demographically distinct sites.

Team members engaged in ongoing reflexivity, recognizing how their disciplinary backgrounds spanning clinical informatics, health communication, and SDM shaped analytic attention, interpretation, and theme development. Consistent with contemporary guidance for thematic analysis [36], these practices were intended to enhance analytic depth, transparency, and reflexive rigor.

### Development Process

The development began with an initial low-fidelity wireframe created using Balsamiq [37], a wireframing software. These low-fidelity prototypes presented broad design concepts with minimal detail [38] and were informed by insights from our prior study [11]. The foundational design was established through collaborative sessions with key stakeholders, including cancer communication experts.

Once the initial design and content were determined, we developed a high-fidelity interactive prototype using Adobe XD [39], which closely replicated the visual design and interactive features of the intended final tool [38]. The final tool was organized into 4 main sections: a brief introduction, educational content on LCS, personalized risk information, and a concluding segment outlining the next steps.

Based on these prototypes, we developed a fully functional web application called MyLungHealth, available in both Spanish and English. The Spanish version was developed using the University of Utah’s foreign language translation service, which provided professional translations of all tool content and associated materials. Throughout the focus groups and interviews, participants were shown the tool via live demonstrations or screenshots, allowing for interaction and feedback.

### Study Settings

Recruitment was conducted from primary care patient populations at 2 sites: UHealth and NYULH. This dual-site approach was intentionally designed to capture diverse patient perspectives. UHealth serves a broad patient population across the Intermountain West region, including rural and underserved communities, while NYULH serves a large urban population in the New York City metropolitan area with diverse socioeconomic and cultural backgrounds.

### Recruitment

Participants were identified using EHR data from both institutions. Inclusion criteria followed the US Preventive Services Task Force guidelines for LCS [3], targeting adults aged 50 to 80 years with a 20+ pack-year smoking history who either currently smoke or have quit within the last 15 years. Spanish-speaking individuals were intentionally recruited for interviews to review the Spanish translation of the educational tool.

Recruitment was conducted using PHR messages at NYULH and email messages at UHealth, which were linked to a screening survey hosted in REDCap (Research Electronic Data Capture) [40,41] to assess interest and eligibility. The REDCap survey included a consent cover letter. Eligible participants were initially invited to focus groups. As the intervention matured to a stage where significant changes were no longer being made in response to focus group feedback, subsequent invitations were extended to both prior focus group participants and new participants for individual interviews.

### Data Collection

We employed multiple qualitative assessment methods, including think-aloud protocols with contextual inquiry [42,43] and critical incident technique interviews, [44,45] in both focus groups and individual interviews.

#### Focus Groups

Focus groups were conducted via Zoom and followed a semistructured format [46,47]. Each session began with an introduction of the interviewers, an explanation of the study’s purpose and research goals, an overview of LCS, and an explanation of the tool’s role in informed decision-making. Participants were guided through features of the MyLungHealth prototype to stimulate discussion on usability, content, and functionality. No prior relationship existed between the researchers and the participants. Only researchers and participants of the study were present for these focus groups.

The focus group script was created in collaboration with health communication experts and included a structured introduction, ground rules, and prepared questions to ensure data comparability across sessions. Questions explored participants’ views on lung cancer, experiences discussing smoking with health care providers, and reactions to the educational tool and its personalized features, such as risk data and visualizations.

Each focus group session lasted approximately 2 hours. Sessions continued until thematic saturation was reached, with no new themes or insights emerging from the discussions. Feedback from each session was collectively reviewed by the research team, and modifications to MyLungHealth were implemented based on group consensus and discussion before the next session.

#### Interviews

Once MyLungHealth was deemed nearly ready for clinical deployment, we conducted in-depth, one-on-one interviews to gain deeper insights into individual patient experiences with LCS and their preferences regarding SDM. These interviews lasted approximately 60 minutes and began with an introduction of the interviewers, an explanation of the study’s purpose and research goals, and an overview of LCS. Similar to the focus groups, no prior relationship existed between the researchers and the participants. Only researchers and participants of the study were present during these interviews.

Using the critical incident technique [44], we encouraged participants to reflect on their prior experiences with LCS and provider discussions, as well as to provide detailed feedback on the design and usability of MyLungHealth.

The interviews allowed for deeper exploration of issues and more extensive interaction with the tool than was possible in a group context. We intentionally recruited participants who might be able to review the Spanish-language version of MyLungHealth after all materials had been translated. To assess whether participants were comfortable participating in Spanish, we asked two screening questions: (1) “Do you feel comfortable providing feedback on educational materials written in Spanish?” and (2) “Do you feel comfortable providing feedback on a Spanish-language educational video?” Eight participants responded “yes” to the first question, and 7 responded “yes” to the second; 1 participant indicated discomfort with reviewing a Spanish-language video. These screening questions served as the sole measure of Spanish-language comfort, and no additional self-report of Spanish-speaking ability was collected.

However, when the Spanish materials were presented during the interview, 7 of these individuals declined, explaining that although they could understand conversational Spanish, they lacked confidence with medical terminology or did not feel fluent enough to provide detailed feedback. As a result, only 1 participant chose to continue the interview in Spanish and provided feedback on the translated tool. Participants self-determined whether their Spanish proficiency was adequate for this task.

Focus group and interview participants were recruited separately using the same eligibility criteria. Prior focus group participants were also invited to participate in interviews, though only one accepted. This individual is counted once in the total unique participant count (n=51).

### Ethical Considerations

This study protocol was approved by the institutional review boards (IRBs) of UHealth and NYULH under a single IRB (IRB_00153806), with the UHealth IRB serving as the IRB of record. All participants provided informed consent prior to participation, and participant privacy and confidentiality were maintained. Participants received a US $50 gift card as compensation for their participation.

### Data Analysis

We applied Braun and Clarke’s principles for thematic analysis to examine the data, as this method is well-suited for drawing out views, opinions, experiences, and values [35,36]. All focus group and interview discussions were audio-recorded, transcribed verbatim, and deidentified for analysis. Field notes were also taken during the focus group and interview sessions and used to inform iterative design modifications to the tool. Anonymized transcripts were securely stored and uploaded to Dedoose (SocioCultural Research Consultants, LLC), a qualitative data analysis platform [48].

Our coding approach combined a hybrid inductive-deductive method. Initially, we applied deductive codes based on the existing codebook from our prior study [11]. We then used an inductive process to develop additional codes for capturing emerging themes. Four team members with expertise in qualitative research, health care, and SDM collaborated in the coding process to ensure reflexive interpretation and minimize bias.

Regular team meetings were held to ensure consistent application of codes, refine definitions, and resolve discrepancies through discussion and consensus [36]. As the analysis progressed, the codebook was refined to identify new patterns and insights directly from the data. Once alignment was achieved among coders, the remaining transcripts were analyzed independently, with ongoing meetings to address emerging issues. At the conclusion of coding, all applied codes were reviewed collectively to ensure consensus, and similar codes were then synthesized into broader themes.

### Iterative Refinement

Between each focus group and before subsequent sessions, the research team discussed participant feedback and assessed it for feasibility, work effort, and appropriateness. These team discussions, rather than the formal thematic analysis, guided the immediate refinements to the MyLungHealth prototype. When feedback was clear and consistent with an obvious course of action, modifications were implemented before the next session. When feedback was more nuanced or conflicting, the research team reached a group consensus on whether and how to address the feedback. This real-time iterative approach allowed for rapid refinement based on user input while ensuring that changes aligned with the tool’s objectives and technical constraints. The formal thematic analysis described above was conducted after data collection was complete and served to identify broader patterns and insights across the entire development process, rather than driving the interim design modifications.

## Results

### Recruitment

A total of 51 unique participants were recruited between May and December 2023: 24 from UHealth and 27 from NYULH. We conducted 7 focus groups with 34 participants and 18 individual interviews. One participant contributed to both a focus group and an interview. Participant demographic characteristics are summarized in Table 1. In the final stages of data collection, no novel themes emerged from either focus groups or interviews, confirming that thematic saturation had been achieved.

**Table 1.** Participant demographic characteristics of focus groups and interviews (N=51).

Characteristic	Value, n (%)
Gender	
Female	32 (63)
Male	19 (37)
Race	
White	34 (67)
Black or African American	16 (31)
Asian	2 (4)
Native Hawaiian or other Pacific Islander	0 (0)
American Indian or Alaska Native	2 (4)
Other	0 (0)
Ethnicity	
Hispanic or Latino	4 (8)
Not Hispanic or Latino	47 (92)
Spanish speaking	
Yes	1 (2)
Limited^a^	7 (14)
No	43 (84)
Technology comfort level	
Very comfortable	40 (78)
Somewhat comfortable	10 (20)
Slightly comfortable	1 (2)
Education level	
More than a 4-year college degree (graduate or professional degree)	9 (18)
4-year college graduate	17 (33)
Some college or 2-year degree	19 (37)
High school graduate or GED^b^	4 (8)
Some high school but did not graduate	2 (4)

a Limited Spanish speakers are defined as individuals who self-reported feeling comfortable providing feedback on the Spanish-language version of the tool but, when presented with the Spanish materials during the interview, declined to do so, explaining that although they could understand conversational Spanish, they lacked confidence with medical terminology or did not feel fluent enough to provide detailed feedback.

b GED: general educational development.

### Thematic Analysis of User Feedback

Our analysis of focus group and interview data revealed 6 major themes related to participants’ experiences and preferences regarding the MyLungHealth decision aid. Table 2 presents these themes, their associated subthemes, and representative participant quotes.

**Table 2.** Themes, subthemes, and representative quotes from participant feedback on the MyLungHealth decision aid.

Theme and subtheme	Representative quote
Domain—interpretation and impact of personalized risk information	
Theme 1: participants struggled to interpret numeric personalized risk information due to this information evoking emotional responses, comprehension difficulties, and lack of contextualization	
Numerical risk predictions evoke emotional responses including anxiety and fear	“They have a 7% in the next five years, if they keep smoking, to develop cancer and 20% chance of dying from cancer if they don’t stop. So both of them are scary when you think of it for yourself.” “That’s kind of scary… it might scare people into doing something, and it might scare people into not wanting to do anything at all.”
Challenges in comprehending and contextualizing numerical percentage risks	“Yeah. That seems really low. I agree. I have a 2% chance of getting into an accident on the way to work. I’m still going to drive to work.”
Need for transparency about data sources and risk calculation methods	“Where did those numbers come from? Would it be possible to throw a link down at the bottom that says, ‘For more information about how we calculate this risk... click here’?” “The 7% chance of developing lung cancer, people need to see where that actual data came from.”
Preference for focusing on screening benefits rather than cancer risk	“You should tell them what benefits they would get out of the screening rather than what their percentage of developing lung cancer would be.”
Theme 2: personalized risk information influenced motivation and behavior change differently across participants, depending on how they perceived the potential benefits of screening and quality of life changes	
Personalized risk information as a potential motivator for action	“I think it would bring some action... Actually seeing it probably would be a motivator for me.”
Resistance to change when perceived benefits appear minimal	“Why bother for one more year?” “If you’re 85, and you’re going to live 3.4 years longer, it’s like, ‘Well, what’s the point?’”
Domain—autonomy, privacy, and user interface preferences	
Theme 3: participants valued autonomy and control over their personal health data, including the ability to edit information, explore scenarios, and had data privacy concerns	
Importance of being able to edit and correct personal health information	“I like that you can change the answers if they’re wrong. I hate it on medical things if something’s wrong and I can’t change it.”
Appreciation for interactive features that demonstrate how behavior changes affect risk	“I like that you can kind of play around with it and see what would happen, how your risk would change if you did quit for five years.”
Concerns about privacy and disclosure of smoking history	“People are embarrassed about their smoking history, and they feel – I don’t know the extent of how truthful people are about it. And they’re being judged or – it’s not a simple thing to get people to really come clean.” “One of my friends, she’s a secret smoker, has smoked as long as I have. And she has always told her doctor she doesn’t smoke.”
Theme 4: participants preferred user interfaces with straightforward language, intuitive visual elements, and multiple format options for information consumption	
Positive response to straightforward language and presentation	“It’s straightforward. I don’t see anything wrong with the way it was worded. It’s to the point.”
Initial confusion with visual risk scale representation	“Would you be clicking on that, or what is that line chart?... Oh, I get it. So it’ll give you your risk score?”
Appreciation for multiple information format options (video and text)	“I like the fact that it offers the educational information in both video and text... I always prefer to read the information so I can digest it and apply critical thinking.” “I’m excited about the video, but I like the fact that it’s video and text. So you have the option to watch the video or read through.”
Domain—integration with clinical workflows and patient portal systems	
Theme 5: participants expected integration between the decision aid and health care provider workflows, including automated communication and convenient follow-up options	
Desire for communication of tool use to health care providers	“So I’m anticipating this would be information that goes back into the electronic medical record and may alert the primary care provider who has seven million [things] that they have to cover at your next appointment.”
Desire for convenient follow-up action features	“Would you like to schedule a screening now? Yes/no? And then it could bring up a calendar with the dates that are available.”
Frustration with perceived lack of integration between decision aids and provider care	“It doesn’t seem like a lot of these online tools actually get into the practitioner’s head.”
Theme 6: participants expressed mixed experiences with PHR^a^ systems but generally appreciated their convenience and the decision aid’s integration with the PHR	
Appreciation for tool integration with existing PHR systems	“But again, the approach of having it in your MyChart, for me, really sets a level of care. So I think that would cause someone to be like, ‘Oh, wow. Well, let me look at that. Let me check that out.’”
Positive perceptions of PHR convenience for health care management	“MyChart is a very, very handy tool. That’s how we keep track of everything. Appointments, the whole nine yards. Too bad the other hospitals don’t have the same thing.” “I love MyChart. I absolutely love it. I think it’s the best thing ever. I think for the people that use it, this needs to be on there for people who use it.”
Frustration with redundant data collection and perceived provider inattention to PHR data	“What really bothers me most though is the fact that I still get the very same questionnaire on paper even though I’ve got all this information in MyChart.” “The pre-visit checklists are a pain… I don’t do them because my doctor’s the same way. She asks me all the questions again, anyway.”

a PHR: personal health record.

Our thematic analysis identified 6 key themes that characterized participants’ experiences with the MyLungHealth decision aid, as shown in Table 2. These 6 themes were organized into three overarching domains: (1) interpretation and impact of personalized risk information; (2) autonomy, privacy, and user interface preferences; and (3) integration with clinical workflows and patient portal systems.

### Domain 1: Interpretation and Impact of Personalized Risk Information

#### Theme 1: Challenges in Interpreting Personalized Risk Information

Participants struggled to interpret personalized risk information, with many finding numerical risk predictions simultaneously informative and anxiety-provoking. Participants frequently had difficulty contextualizing percentage-based risk figures, often drawing incorrect comparisons between their lung cancer risk and other everyday activities. Some participants expressed a desire for additional information about data sources and calculation methods.

#### Theme 2: Influence of Risk Information on Motivation and Behavior Change

Personalized risk information influenced motivation and behavior change differently across participants. While some found personalized risk information motivating for taking action, others expressed resistance when perceived benefits appeared minimal, particularly regarding potential years of life gained through screening. This was found to be especially true when participants felt that quality of life would be poor during the potential gained life years.

### Domain 2: Autonomy, Privacy, and User Interface Preferences

#### Theme 3: Autonomy and Privacy in Health Data Management

Participants valued autonomy and control over their personal health data, appreciating features that allowed them to edit their smoking history information and explore how behavioral changes might affect their risk levels. Some participants expressed privacy concerns regarding the disclosure of their smoking history, highlighting the sensitive nature of this information.

#### Theme 4: User Interface Preferences and Risk Visualization

Regarding user interface preferences, participants favored straightforward language and clarity in presentation. Initial confusion with the visual risk scale was common, though participants generally appreciated having risk visualizations accompanied by qualitative labels. The visual risk presentation was a significant area of focus in participant feedback, with suggestions for improving color coding and labeling to enhance comprehension.

### Domain 3: Integration With Clinical Workflows and Patient Portal Systems

#### Theme 5: Expectations for Clinical Workflow Integration

Participants expected integration between the decision aid and health care provider workflows. Many anticipated that their tool usage would be automatically communicated to their providers, leading to informed discussions during appointments. Participants also desired convenient follow-up features, such as appointment scheduling capabilities within the tool.

#### Theme 6: Mixed Experiences With PHR Systems

Mixed experiences with PHR systems emerged as an important theme, with participants generally viewing PHR systems positively for health care management while expressing frustration with redundant data collection and the perception that providers did not review the information they entered. Participants valued tools that integrated with existing PHR platforms but wanted assurance that providers would acknowledge their engagement with these tools during their upcoming visits.

### Design Changes Based on User Feedback

Based on participant feedback, several significant design modifications were made to the MyLungHealth decision aid during the iterative development process. Table 3 presents the key interface elements that were modified and the specific changes implemented in response to user feedback.

**Table 3.** Interface elements and design modifications to MyLungHealth based on user feedback.

Interface element	Qualitative domain	Design modification based on user feedback
Risk presentation screen	Interpretation and impact of personalized risk information	Simplified the information-dense risk presentation from the initial prototype (Figure 1A) to a cleaner final design (Figure 1B), moving detailed numerical risk information into an expandable “More Information” section that users can access on demand
Information delivery format	Autonomy, privacy, and user interface preferences	Implemented both video and text options for educational content (Figure 2), allowing users to choose their preferred format for consuming information
Provider notification	Integration with clinical workflows and patient portal systems	Created an EHR^a^ alert (Figure 3) to notify clinicians when patients have used MyLungHealth, facilitating integration with provider workflows

a EHR: electronic health record.

**Figure 1.**
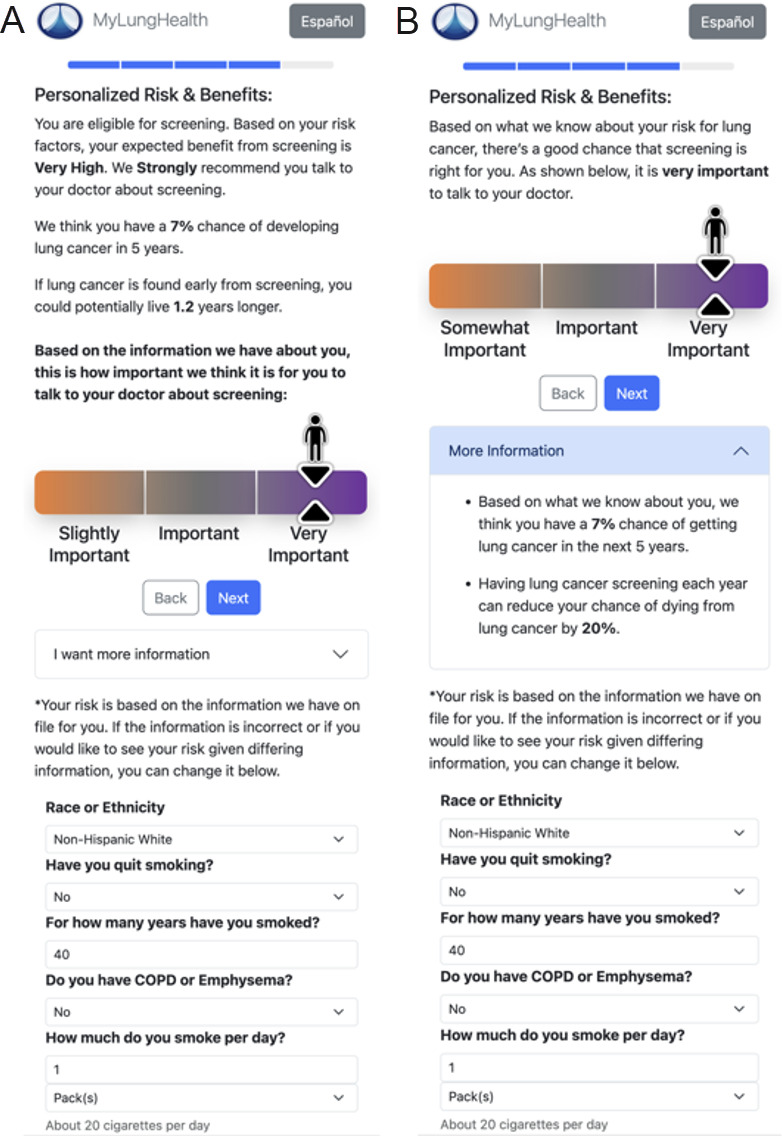
Risk presentation screen in MyLungHealth. (A) Risk details that were prominently displayed in the prototype were moved into (B) an optional “More Information” section in the final design.

**Figure 2.**
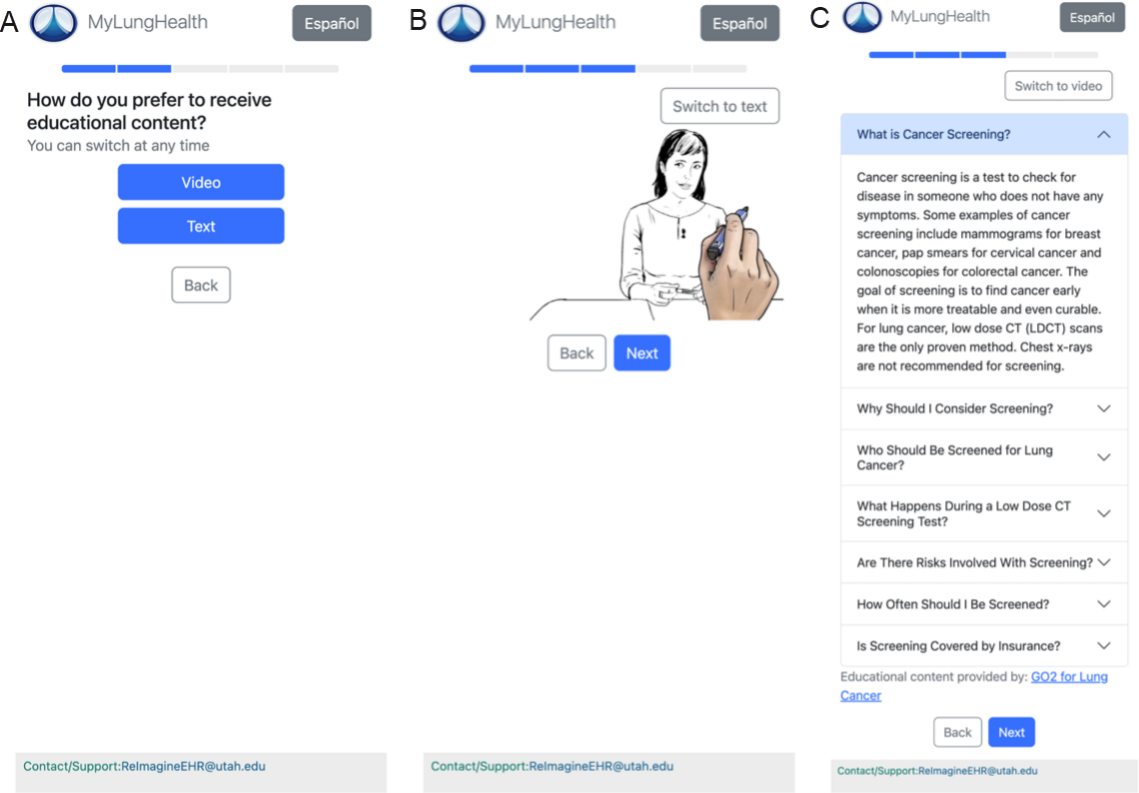
Screenshots demonstrating multimodal support for patient educational materials in MyLungHealth to accommodate individual patient preferences. (A) Patients are offered a choice of format for viewing the educational material, selecting either (B) video or (C) text. Patients can switch between formats at any time using a button located in the top-right corner.

**Figure 3.**
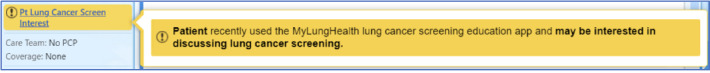
An electronic health record alert notifying the clinician that the patient has engaged with MyLungHealth.

The design changes focused on addressing key user preferences and concerns identified during focus groups and interviews. To enhance information accessibility, the risk presentation screen was substantially simplified, with detailed risk information moved to optional expandable sections that users could access if desired (Figure 1). This change was made in response to participant feedback about confusion and anxiety related to numerical risk data.

To accommodate different learning preferences, educational content was made available in both video and text formats, allowing users to engage with the material in their preferred way (Figure 2). To address participant concerns about provider awareness of their tool usage, we implemented an EHR alert system to notify clinicians when patients had accessed MyLungHealth (Figure 3).

These design modifications reflect the iterative, user-centered development process and demonstrate how participant feedback directly informed the evolution of the MyLungHealth decision aid from the initial prototype to final implementation.

## Discussion

### Overview of Findings

In this study, the iterative design of MyLungHealth, a patient-centered LCS education tool, was informed by extensive user feedback gathered through 7 focus groups with 34 participants and 18 individual interviews. In a follow-up randomized clinical trial, the MyLungHealth tool was shown to significantly improve LCS ordering and collection of smoking information [49].

The thematic analysis of this feedback provided critical insights that can inform the development of future health care decision aids. Our findings are organized into three domains: (1) interpretation and impact of personalized risk information; (2) autonomy, privacy, and user interface preferences; and (3) integration with clinical workflows and patient portal systems.

### Interpretation and Impact of Personalized Risk Information

A central theme across our findings concerned how participants interpreted and responded to personalized risk information. We found that presenting numerical risk information requires careful consideration, as some users found such data informative and motivational, while others found it difficult to understand, anxiety-provoking, or uncompelling. This observation aligns with our prior focus group study [11], a usability analysis conducted on similar web-based LCS decision aids for veteran populations [22], and a survey and focus group study on patients’ attitudes toward LCS and decision aids, which found that the magnitude of patients’ lung cancer risk and benefits was lower than they expected [10]. To address this concern, we relocated detailed numerical risk information to an optional “More Information” section, simplifying the presentation while still making comprehensive data available to those who desired it. These design decisions reflect a broader tension in risk communication: personalized risk estimates have the potential to motivate screening behavior, but their impact depends on how well they are understood and received by diverse patient populations.

### Autonomy, Privacy, and User Interface Preferences

Participants also consistently expressed a preference for simplicity in information presentation, which aligns with recommendations for simplified graphic presentation to enable effective communication [50], as well as recommendations for the design of decision aids by Fagerlin et al [51]. With respect to autonomy, participants valued the ability to update their smoking history within the tool, which has been associated with increased patient engagement and satisfaction [52]. However, this feature raised concerns about data privacy. Emphasizing that personal information would remain confidential and accessible only to their care team may help alleviate these concerns, suggesting that transparent messaging about data usage and privacy represents an area for potential improvement in building user trust.

These preferences for autonomy and privacy extended to how participants wanted information presented within the interface itself. Prior studies also found that patients had issues with navigating decision aids [22], so we ensured that MyLungHealth had a linear flow with a short introduction (education in either video or text form), presentation of risk information, and next steps. Following these best practices, we prioritized presenting less information rather than overwhelming patients with excessive details and offered risk information in both a numerical risk format and through visual aids.

### Integration With Clinical Workflows and Patient Portal Systems

Finally, participants highlighted the importance of integrating patient-facing tools into existing clinical workflows and patient portal systems. Participants expressed a strong desire for actionable follow-up options integrated within MyLungHealth, particularly direct scheduling and reminders. Although direct scheduling without clinician involvement is not feasible, given the need for SDM, this feedback highlights patients’ desire for a more seamless screening process. The implementation of an EHR alert addresses this need by notifying providers when patients use MyLungHealth, helping to integrate the tool into a standard clinical workflow rather than creating an additional step that might be bypassed. Similar to how integration into the patient portal may increase utilization [53], the alert system may increase provider involvement and engagement in SDM, creating a more comprehensive approach to screening discussions.

MyLungHealth is a Health Level Seven International Substitutable Medical Applications Reusable Technologies (SMART) [54] on the Fast Healthcare Interoperability Resources (FHIR) [55] application distributed via previsit questionnaires from the patient portal. MyLungHealth follows the SIMPLE (Standards-based Implementation Maximizing Portability Leveraging the EHR) architectural pattern for integrating patient-facing apps into clinical workflows [56]. By leveraging the Health Level Seven International SMART and FHIR standards, SIMPLE allows seamless application integration into the PHR, without requiring additional software installation, ensuring that the integration supports UCD through its streamlined, user-friendly nature that maximizes intervention usability by the target population.

A strength of this study is the substantial and diverse participant pool, which allowed us to reach saturation for insights. Notably, 24 (47%) participants came from racial or ethnic minority backgrounds, enhancing the generalizability of our findings to diverse populations.

### Limitations

This study has limitations. We recruited only participants who were comfortable engaging in focus groups or interviews via Zoom, potentially skewing our findings toward individuals with higher digital literacy and health literacy. However, this sample selection was appropriate for our intervention, as MyLungHealth requires PHR access for use. Additionally, the study was conducted at only 2 academic medical centers, which may limit generalizability. Nevertheless, the inclusion of sites from different regions (Northeast and Mountain West), including a highly diverse area (New York City), strengthens the applicability of our findings.

The insights gained from this study have supported the iterative enhancement of MyLungHealth in a multi-institutional patient-randomized controlled trial at UHealth and NYULH to assess its clinical impact on LCS rates and patient engagement [34].

Additionally, although participants were screened for comfort in reviewing Spanish-language materials to evaluate the translated version of the tool, only 1 participant ultimately completed an interview using the Spanish-language version. While 7 additional participants indicated initial comfort engaging with Spanish-language educational content during screening, they ultimately declined to do so, explaining that, although they could understand conversational Spanish, they lacked confidence with medical terminology or did not feel fluent enough to provide detailed feedback. As a result, feedback from the single Spanish-language interview was insufficient to identify themes specific to Spanish-speaking users or to independently guide substantial modifications to the translated version. Instead, design changes identified through feedback from English-speaking participants were applied to both language versions. To maintain accuracy, the University of Utah’s foreign language translation service updated the Spanish-language materials after each iterative modification to ensure revisions were appropriately reflected in the translated tool. Future studies with larger samples of Spanish-speaking participants are needed to assess language-specific usability considerations, comprehension of medical terminology, and cultural appropriateness of the tool.

### Conclusions

This study reports on the user-centered development process of MyLungHealth, a patient-facing educational tool designed to enhance SDM for LCS. Through focus groups and interviews with diverse participants from 2 health care systems, we identified 6 major themes reflecting user experiences and preferences: challenges with risk information interpretation, variable impact of personalized risk data on motivation, desire for autonomy over health data, preference for straightforward interfaces with multiple format options, expectations for integration with clinical workflows, and mixed experiences with patient portal systems. These insights aligned with prior findings and recommendations by ourselves [11] and others [10,22,50,51] and directly informed iterative design modifications, including simplified risk presentation, multimodal educational content options, and provider notification features.

The findings highlight important considerations for developing patient-centered health care decision aids. Effective tools must balance providing comprehensive information with preventing cognitive overload, offer appropriate contextualization of risk data, ensure seamless integration with clinical workflows, and maintain user control over personal health information. By implementing these design principles within an interoperable SMART on FHIR architecture, MyLungHealth exemplifies how patient-facing applications can be effectively integrated into digital health infrastructure while supporting meaningful SDM. The recently completed randomized controlled trial provides evidence regarding the tool's impact, potentially offering a model for similar interventions addressing other preventive services [49].

## Abbreviations

EHR: electronic health record
FHIR: Fast Healthcare Interoperability Resources
IRB: institutional review board
LCS: lung cancer screening
LDCT: low-dose computed tomography
NYULH: New York University Langone Health
PHR: personal health record
REDCap: Research Electronic Data Capture
SDM: shared decision-making
SIMPLE: Standards-based Implementation Maximizing Portability Leveraging the EHR
SMART: Substitutable Medical Applications Reusable Technologies
UCD: user-centered design
UHealth: University of Utah Health
